# Inverse differential quadrature method: mathematical formulation and error analysis

**DOI:** 10.1098/rspa.2020.0815

**Published:** 2021-04

**Authors:** Saheed O. Ojo, Luan C. Trinh, Hasan M. Khalid, Paul M. Weaver

**Affiliations:** Bernal Institute, School of Engineering, University of Limerick, V94 T9PX, Castletroy, Ireland

**Keywords:** numerical analysis, high-order differential equations, direct approximation, inverse differential quadrature method, error analysis, numerical stability

## Abstract

Engineering systems are typically governed by systems of high-order differential equations which require efficient numerical methods to provide reliable solutions, subject to imposed constraints. The conventional approach by direct approximation of system variables can potentially incur considerable error due to high sensitivity of high-order numerical differentiation to noise, thus necessitating improved techniques which can better satisfy the requirements of numerical accuracy desirable in solution of high-order systems. To this end, a novel inverse differential quadrature method (iDQM) is proposed for approximation of engineering systems. A detailed formulation of iDQM based on integration and DQM inversion is developed separately for approximation of arbitrary low-order functions from higher derivatives. Error formulation is further developed to evaluate the performance of the proposed method, whereas the accuracy through convergence, robustness and numerical stability is presented through articulation of two unique concepts of the iDQM scheme, known as Mixed iDQM and Full iDQM. By benchmarking iDQM solutions of high-order differential equations of linear and nonlinear systems drawn from heat transfer and mechanics problems against exact and DQM solutions, it is demonstrated that iDQM approximation is robust to furnish accurate solutions without losing computational efficiency, and offer superior numerical stability over DQM solutions.

## 1. Introduction

Engineering systems are typically governed by complex high-order differential equations which require numerical methods to provide accurate solutions. To approximate such systems, the domain of interest is discretized by means of interpolation (shape) functions and its higher derivatives, which are defined over a subdomain of interest called elements. Examples of methods available in this context are element-based methods such as finite-element method [1–3], boundary element method [4,5], finite difference method [6–8] or finite volume method [9,10]. On the other hand, a class of high-order mesh-free methods such as radial basis function networks (RBFN) [11–13], element-free Galerkin method [14–16], diffuse element method [17], or high-order collocation methods such as the Chebyshev method [18–20] and the differential quadrature method (DQM) [21–27], have been widely applied for solving engineering and science problems. In all of these methods, target derivatives of an arbitrary function are directly approximated as a weighted sum of the function in the domain. According to Mai-Duy & Tran-Cong [28], in the process of differentiation, errors of function approximation may amplify significantly as influenced by local effects of the approximant. With respect to analysis of engineering structures, such error amplification can affect the accuracy of high-order secondary variables including strains, stresses, moments, and shear forces. To this effect, indirect radial basis function networks (IRBFN) were proposed in [29]. In contrast to the RBFN approach, IRBFN approximate a derivative function using RBFN and then recover the original function by integration, which is less sensitive to noise.

DQM as proposed by Bellman and others [21,22] has received widespread attention in the research community due to spectral accuracy and fast convergence that make it desirable for vast engineering applications. For example, in the field of structural mechanics, the DQM approach has been explored for static analysis [30–32], free vibration analysis [33–35], buckling analysis [29,36] and large deflection post-buckling analysis [37]. In the context of fluid mechanics applications, DQM has been widely applied to obtain solutions of convection problems [38–40] and Navier–Stokes equations [41,42], heat transfer analysis [43,44], and magnetohydrodynamic duct flow problems [45]. It is instructive to note that in [38–40,42] the so-called localized DQM is adopted in combination with RBF since this strategy allows versatility in using meshes or meshless systems [46]. Financial engineering is another area where the merits of DQM have been explored for computational analysis [47].

To generalize DQM for engineering applications, Shu [23] proposed a general approach which admits the use of different base polynomials for computation of DQM weights. According to Shu’s approach [23], computation of the differential quadrature (DQ) weights for first-order and higher-order derivatives can be generalized by appropriate choice of base vectors in the linear vector space. Furthermore, Quan & Chang [48] as well as Wang [49] contended that explicit formulation of the weighting coefficients by using test functions can overcome the ill-conditioning arising from a large number of grid points. In this regard, to allow for explicit computation of the DQ weights, setting the base vectors as base polynomials (such as Lagrange polynomials, Chebyshev polynomials or Legendre polynomials) in a linear vector space in the so-called polynomial-based differential quadrature (PDQ) method is recommended [23,48,49]. To mitigate the sensitivity of DQM solutions to grid spacing to the Runge phenomenon, Wang [49] noted that non-uniform grid distribution is required to obtain reliable solutions. Readers are referred to Wang [49] for classical examples of non-uniform grid distributions that guarantee good accuracy, numerical stability and fast convergence. On the other hand, for applications like wave propagation in space where uniformly distributed grid points are required for accurate numerical estimation, the localized DQM (LDQM), which applies DQM approximations within a small neighbourhood of the point of interest, is crucial to keep the balance between the accuracy and stability of the numerical estimates [25]. In recent times, remarkably in computational fluid mechanics applications, the so-called RBF-based DQ method, as well as the LDQM variant, are increasingly being adopted to extend the merits of the DQM, as already noted in [38–40,42]. For the purpose of the current work, the PDQ-based approach with a non-uniform grid structure is adopted.

Despite the positive contributions of DQM to various engineering fields, application to high-order systems may suffer from numerical inaccuracy and instability on account of error accumulation in the process of differentiation. According to error analysis outlined in Shu [23], the process of differentiation via DQM may incur multiple orders of inaccuracy which increasingly deteriorates for higher-order approximations, especially at the domain boundaries. Relying on the gains of IRBFN, Wu & Ren [50] proposed a ‘differential quadrature method based on approximation of the highest derivative’ (DQIHD) to reformulate some engineering problems which yielded accurate solutions. Like IRBFN, and in contrast to DQM, DQIHD approximates the highest derivative in a system and obtains lower-order functions by integration of the high-order primary estimate. Although Wu & Ren [50] associated this method, i.e. DQIHD, with DQM, we cannot find any empirical relationship between DQIHD and DQM apart from the use of Lagrange polynomials for computation of weights, as also done by DQM. Moreover, the two methods use different routines to determine the weights, and the properties of resulting matrices for DQM and DQIHD are fundamentally different. In addition to this, Wu & Ren [50] did not provide comparative analysis with DQM to demonstrate the performance of DQIHD. As a result, given the DQM scheme, which primarily estimates the lowest order function in a system, and the DQIHD scheme, which primarily estimates the highest order function in a system, it is not apparent which is the best approach to adopt for system solutions.

Consistent with the idea of indirect approximation, we propose a novel inverse differential quadrature method (iDQM) for system approximation. The proposed iDQM formulation provides a general framework for approximating arbitrary functions of any order in a system and either recover lower-order functions from high-order functions by iDQM operations or obtain high-order functions from lower-order ones by DQM operations. In this context, it is possible to combine the advantages of low-order numerical differentiation and low-order numerical integration to achieve improved results. A specific case of the proposed iDQM scheme is DQIHD in which the highest order derivative in a system is approximated.

Firstly, a formulation for approximation of arbitrary low-order functions from higher derivatives (not necessarily the highest order as in DQIHD) is derived in §2. To circumvent issues arising from computational inefficiency, associated with analytical integration or numerical complexity due to Gaussian integration, we employ a unique strategy to extract iDQM weights by inversion of existing DQM formula, leading to a robust and efficient routine described in §2.d, which can admit the use of different base polynomials, like DQM. Then, in §3., formulations of error estimates are developed for *iDQM-by-integration* and *iDQM-by-inversion*, which are subsequently compared with DQM error estimates developed by Shu [23]. Section 4. is dedicated to demonstrating the accuracy of the proposed method through basic implementation of iDQM for functional approximation as well as solution of high-order ordinary and partial differential equations representing linear and nonlinear systems, with examples taken from heat transfer and mechanics fields. This process eventually leads to the establishment of concepts of mixed iDQM (MiDQM) and full iDQM (FiDQM) described in §4.b. Consequently, a comprehensive error analysis entailing convergence, robustness, and numerical stability of iDQM operation is illustrated with a boundary value example in §4.g and §4.h while §5. is dedicated to characterizing the computational efficiency of iDQM. Finally, conclusions of the present study are considered in §6.

### (a) Brief introduction to differential quadrature method formulation

According to Weierstrass’s first theorem [21], suppose *f*(*ξ*) is a real valued continuous function defined in a closed interval *ξ* ∈ [*a* *b*], then there exists a sequence of polynomials *P*_*n*_ (*ξ*) which converge to *f*(*ξ*) as *n* tends to infinity. Therefore, if *f*(*ξ*) represents the solution of a partial differential equation, then it can be approximated by a polynomial of a degree less than *N* through the mathematical relation,

1.1 $$f(\xi )\approx P_{n}(\xi )=\sum_{k=0}^{N-1}c_{k}\xi^{k},$$

where *c*_*k*_ represents constant weights to be determined and *ξ*^*k*^ are the linearly independent basis vectors in the *N*-dimensional linear vector space, *V*_*N*_. Adopting Shu’s general approach [23], some sets of base polynomials can be selected to determine the weights, *c*_*k*_. Furthermore, according to Shu [23], the numerical difficulty of determining the weights as *N* becomes large can be eliminated by considering Lagrange interpolation polynomials as basis vectors in which *P*_*n*_ (*ξ*) is then approximated by

1.2 $$P_{n}(\xi )=\sum_{i=1}^{N}f_{i}l_{i}(\xi )+E(\xi ),$$

where *E*(*ξ*) is the polynomial approximation error, and *l*_*i*_ (*ξ*) is a (*N* − 1) degree Lagrange polynomial defined as

1.3 $$l_{i}(\xi )=\frac{M(\xi )}{M^{\left(1\right)}(\xi_{i})(\xi -\xi_{i})},$$

where $M(\xi )=\prod_{i=1}^{N}(\xi -\xi_{i}),$ and $M^{\left(1\right)}(\xi_{i})=\prod_{k=1,k\neq i}^{N}(\xi_{i}-\xi_{k}),$ and *f*(*ξ*_*i*_) is the functional value at a discrete point *i*. In line with DQM routines outlined in [23], the first derivative of the function *f*(*ξ*), $f_{i}^{\left(1\right)}$, at a discrete point *i* in the domain *ξ* ∈ [*a* *b*] can be realized as follows,

1.4 $$f_{i}^{\left(1\right)}=\sum_{j=1}^{N}a_{ij}^{\left(1\right)}f_{j}+E^{\text{dif}(1)}(\xi_{i}),\;\text{for}\;i,j=1,\ldots ,N,$$

where $a_{ij}^{\left(1\right)}=(M^{\left(1\right)}(\xi_{i})/M^{\left(1\right)})(\xi_{j})(\xi_{i}-\xi_{j}),$ and $a_{ii}^{\left(1\right)}=-\sum_{j=1,j\neq i}^{N}a_{ij}^{\left(1\right)}$, for *i* = *j*.

In equation (1.4), *E*^dif(1)^(*ξ*) is the approximation error due to first-order numerical differentiation of *f*(*ξ*), which is given in [23] as

1.5 $$E^{\text{dif}(1)}(\xi )\leq \frac{KM^{\left(1\right)}(\xi )}{N!},$$

where *K* is a constant implied from Shu [23]. For an arbitrary derivative of order *m*, the DQM approximation is characterized by a weighting coefficient $a_{ij}^{\left(m\right)}$ which is evaluated based on the recursive formula,

1.6 $$\begin{array}{rl} & a_{ij}^{\left(m\right)}=m\left(a_{ij}^{\left(1\right)}a_{ii}^{\left(m-1\right)}-\frac{a_{ij}^{\left(m-1\right)}}{\xi_{i}-\xi_{j}}\right),\;\text{for}\;i,j=1,\ldots ,\;m=2,3,\ldots ,N-1,\\ & a_{ii}^{\left(m\right)}=-\sum_{j=1,j\neq i}^{N}a_{ij}^{\left(m\right)}. \end{array}$$

In a compact form, the DQM approximation of *m*th-order derivative of *f*(*ξ*) is represented thus

1.7 $${\text{F}}^{\left(m\right)}={\text{D}}^{\left(m\right)}\text{F}+{\text{E}}^{\text{dif}(m)}\;m=2,3,\ldots ,N-1,$$

where ${\text{F}}^{\left(m\right)}\in R^{N\times 1}$ is the *m*th order derivative of the vector function **F** = [*f*_1_, *f*_2_, …, *f*_*N*_]^*T*^, $\text{F}\in R^{N\times 1}$, while ${\text{D}}^{\left(m\right)}\in R^{N\times N}$ represents the DQM coefficient matrix for the *m*th derivative of **F** according to equation (1.4), and **E**^dif(*m*)^ is the approximation error of the *m*th-order numerical differentiation of **F** given as

1.8 $${\text{E}}^{\text{dif}(m)}(\xi )={\left[E^{\text{dif}(m)}(\xi_{1}),E^{\text{dif}(m)}(\xi_{2}),\ldots ,E^{\text{dif}(m)}(\xi_{N})\right]}^{T},\;{\text{E}}^{\text{dif}(m)}\in R^{N\times 1}$$

Typically, **E**^dif(*m*)^ < <**F**^(*m*)^, so equation (1.8) can be rewritten as

1.9 $${\text{F}}^{\left(m\right)}={\text{D}}^{\left(m\right)}\text{F}+\text{I}c_{0}^{\left(m\right)}\;\text{I}={\left[1,1,\ldots ,1\right]}^{T},\;\text{I}\in R^{N\times 1},$$

where $c_{0}^{\left(m\right)}$ is the mean approximation error of the *m*th-order numerical differentiation expressed as

1.10 $$c_{0}^{\left(m\right)}=\frac{1}{N}\sum_{i=1}^{N}E^{\text{dif}(m)}(\xi_{i}).$$

## 2. Inverse differential quadrature method

This section introduces the mathematical formulation for the iDQM. The idea of iDQM involves functional approximation of high-order derivatives of *f*(*ξ*) and the subsequent recovering of low-order derivatives by integration of the high-order derivatives. Different approaches to achieve this aim in a computationally efficient and numerically stable manner are presented in the following.

### (a) First-order inverse differential quadrature method-by-integration

Suppose we let the first derivative of a function *f*(*ξ*), *f*^(1)^ (*ξ*), be approximated for a fixed *N* as in equation (1.2),

2.1 $$f^{\left(1\right)}(\xi )=\sum_{i=1}^{N}f_{i}^{\left(1\right)}l_{i}(\xi )+E_{1}(\xi ),$$

where $f_{i}^{\left(1\right)}$ is the first derivative of *f*(*ξ*) at a discrete point and *E*_1_ (*ξ*) is the polynomial approximation error for *f*^(1)^. The original function *f*(*ξ*) can then be recovered from *f*^(1)^ by integrating equation (2.1) to get

2.2 $$f(\xi )=\int f^{\left(1\right)}(\xi )\;\text{d}\xi =\sum_{i=1}^{N}f_{i}^{\left(1\right)}\underset{H_{i}(\xi )}{\underbrace{\int l_{i}(\xi )d\xi }}+c_{0}+E_{1}^{\text{int}(1)}(\xi ),$$

where *H*_*i*_ (*ξ*) is a *N*th-order polynomial function, *c*_0_ is the constant of integration and $E_{1}^{\text{int}(1)}(\xi )=\int E_{1}(\xi )\;\text{d}\xi$. In a compact form, equation (2.2) gives

2.3 $$\text{F}={\text{H}}^{\left(1\right)}{\text{F}}^{\left(1\right)}+\text{I}c_{0}+{\text{E}}_{1}^{\text{int}(1)},\;\text{F},{\text{F}}^{\left(1\right)},{\text{E}}_{1}^{\text{int}(1)}\in R^{N\times 1},\;{\text{H}}^{\left(1\right)}\in R^{N\times N},$$

where ${\text{F}}^{\left(1\right)}={\left[f_{1}^{\left(1\right)},f_{2}^{\left(1\right)},\ldots ,f_{N}^{\left(1\right)}\right]}^{T}$ and ${\text{E}}_{1}^{\text{int}(1)}={\left[E_{1}^{\text{int}(1)}(\xi_{1}),E_{1}^{\text{int}(1)}(\xi_{2}),\ldots ,E_{1}^{\text{int}(1)}(\xi_{N})\right]}^{T}$. It is convenient to recast equation (2.3) as:

2.4 $$\text{F}={\tilde{\text{H}}}^{\left(1\right)}{\tilde{\text{F}}}^{\left(1\right)}+{\text{E}}_{1}^{\text{int}(1)},\;{\tilde{\text{F}}}^{\left(1\right)}\in R^{(N+1)\times 1},\;{\tilde{\text{H}}}^{\left(1\right)}\in R^{N\times (N+1)},$$

where ${\tilde{\text{H}}}^{\left(1\right)}=[{\text{H}}^{\left(1\right)}\;\text{I}]$ and ${\tilde{\text{F}}}^{\left(1\right)}={\left[{\text{F}}^{\left(1\right)}\;c_{0}\right]}^{T}$.

Equation (2.4) represents the iDQM relation which is used to approximate a function from its first derivative.

### (b) Higher-order inverse differential quadrature method-by-integration

Let the *m*th-order derivative of a continuous function *f*(*ξ*) be *f*^(*m*)^ (*ξ*), which is approximated as in equation (1.2),

2.5 $$f^{\left(m\right)}(\xi )=\sum_{i=1}^{N}f_{i}^{\left(m\right)}(\xi_{i})l_{i}(\xi )+E_{m}(\xi ),\;\text{for}\;m>1.$$

*E*_*m*_(*ξ*) is the polynomial approximation error for *f*^(*m*)^. To obtain *f*^(*m*)^ (*ξ*) from equation (2.5) requires *m*th order integration, which leads to

2.6 $$f(\xi )=\sum_{i=1}^{N}f_{i}^{\left(m\right)}H^{\left(m\right)}(\xi )+f_{c}^{\left(m-1\right)}(\xi )+E_{m}^{\text{int}(m)}(\xi ),$$

where *H*^(*m*)^ is (*N* + *m* − 1)th order polynomial functions containing *m*th order integral of *l*_*i*_ (*ξ*), $E_{m}^{\text{int}(m)}$ is the *m*th-order integral of *E*_*m*_, and $f_{c}^{\left(m-1\right)}$is (*m* − 1)th polynomial functions of integration constants given as

2.7 $$f_{c}^{\left(m-1\right)}=c_{0}+\sum_{k=1}^{m-1}c_{k}\frac{\xi^{k}}{k!}.$$

Note: the subscript and superscript of $E_{m}^{\text{int}(m)}$ refers, respectively, to the order of function, i.e. *f*^(*m*)^, and the order of integration operation.

In consistency with equations (2.2) and (2.3), equation (2.6) can be recast in a compact form as

2.8 $$\text{F}={\tilde{\text{H}}}^{\left(m\right)}{\tilde{\text{F}}}^{\left(m\right)}+{\text{E}}_{m}^{\text{int}(m)},\;{\tilde{\text{F}}}^{\left(m\right)}\in R^{(N+m)\times 1},\;{\tilde{\text{H}}}^{\left(m\right)}\in R^{N\times (N+m)},$$

where ${\tilde{\text{H}}}^{\left(m\right)}=[{\text{H}}^{\left(m\right)}\;(1/(m-1)!)\xi^{m-1}\;(1/(m-2)!)\xi^{m-2}\cdots \text{I}]$, ${\text{H}}^{\left(m\right)}\in R^{N\times N}$, **ξ** = [*ξ*_1_, *ξ*_2_, …, *ξ*_*N*_]^*T*^, $\xi \in R^{N\times 1}$, ${\text{E}}_{m}^{\text{int}(m)}={\left[E_{m}^{\text{int}(m)}(\xi_{1}),E_{m}^{\text{int}(m)}(\xi_{2}),\ldots ,E_{m}^{\text{int}(m)}(\xi_{N})\right]}^{T}$, ${\text{E}}_{m}^{\text{int}(m)}\in R^{N\times 1}$ and ${\tilde{\text{F}}}^{\left(m\right)}={\left[{\text{F}}^{\left(m\right)}\;c_{m-1}c_{m-2}\cdots c_{0}\right]}^{T}$, ${\text{F}}^{\left(m\right)}\in R^{N\times 1}$.

Assuming ${\text{E}}_{m}^{\text{int}(m)}<<\text{F}$, the error term can be dropped from equation (2.8) to get

2.9 $$\text{F}\approx {\tilde{\text{H}}}^{\left(m\right)}{\tilde{\text{F}}}^{\left(m\right)}.$$

### (c) Computational aspects of inverse differential quadrature method-by-integration

The matrix of integral coefficients, **H**^(*m*)^, can be computed by analytical integration, which requires a costly symbolic computational operation in Matlab. Besides, analytical integration may not be feasible for some vector basis functions, making this approach cumbersome and undesirable. On the other hand, Gaussian integration can be used to compute **H**^(*m*)^ efficiently. However, the numerical accuracy and stability of this operation depends on the number of Gauss points, which is typically chosen intuitively. The additional computational variable manifested by Gaussian integration increases the complexity of the *iDQM-by-integration* especially for high-order functions. On this basis, to compute **H**^(*m*)^, we seek a novel alternative that, respectively, resolves the inefficiency and numerical instability bottlenecks of analytical and Gaussian integrations yet sufficiently preserves the accuracy of both methods.

### (d) First-order inverse differential quadrature method-by-inversion

To avoid the deficiency imposed by direct integration or Gaussian integration in the computation of **H**^(*m*)^, we now describe an alternative formulation which is computationally efficient and numerically stable to compute the iDQM coefficient matrix.

Assuming the coefficient matrix, **D**, is invertible, equation (1.7) can be rearranged to make the vector function, **F**, the subject, in case of first order as

2.10 $$\text{F}={\text{D}}^{-1}{\text{F}}^{\left(1\right)}-[{\text{D}}^{\left(-1\right)}{\text{E}}^{\text{dif}(1)}],$$

or alternatively as in equation (1.9),

2.11 $$\text{F}={\bar{\text{D}}}^{\left(1\right)}{\text{F}}^{\left(1\right)}+\text{I}{\bar{c}}_{0},\;\bar{\text{D}}\in R^{N\times N},$$

where ${\bar{\text{D}}}^{\left(1\right)}={\text{D}}^{-1}$ and $\bar{c_{0}}$ is the is the mean error distribution for first-order *iDQM-by-inversion*, which is expressed as

2.12 $$\bar{c_{0}}={\left[-{\bar{\text{D}}}^{\left(1\right)}{\text{E}}^{\text{dif}(1)}\right]}_{\text{mean}}.$$

To compare equation (2.11) with equation (2.3), we consider derivation of the constant $\bar{c_{0}}$ in terms of the integration constant *c*_0_. Assuming that the function *f*(*ξ*), evaluated at point *p* in the closed interval *ξ* ∈ [*a* *b*], *f*(*ξ*_*p*_), approaches the constant *c*_0_ due to the fact that the polynomial function *l*_*i*_ (*ξ*_*p*_) → 0, then

2.13 $$f(\xi_{p})\to c_{0}+E^{\text{int}(1)}(\xi_{p})\;\text{s.t.}\;l_{i}(\xi_{p})\to 0.$$

Now, evaluating *f*(*ξ*_*p*_) using equations (2.3) and (2.11), the following relation can be established,

2.14 $$c_{0}+E^{\text{int}(1)}(\xi_{p})={\left[{\bar{\text{D}}}^{\left(1\right)}\right]}_{p}{\text{F}}^{\left(1\right)}+{\bar{c}}_{0},$$

where ${\left[{\bar{\text{D}}}^{\left(1\right)}\right]}_{p}\in R^{1\times N}$ is the *p*th row vector of matrix ${\bar{\text{D}}}^{\left(1\right)}$. Rearranging equation (2.14) in terms of *c*_0_ gives

2.15 $${\bar{c}}_{0}=c_{0}-{\left[{\bar{\text{D}}}^{\left(1\right)}\right]}_{p}{\text{F}}^{\left(1\right)}+E^{\text{int}(1)}(\xi_{p}).$$

Substituting equation (2.15) into equation (2.11) gives

2.16 $$\text{F}=\underset{{\hat{\text{D}}}^{\left(1\right)}}{\underbrace{\left({\bar{\text{D}}}^{\left(1\right)}-\text{I}{\left[{\bar{\text{D}}}^{\left(1\right)}\right]}_{p}\right)}}{\text{F}}^{\left(1\right)}+\text{I}c_{0}+\text{I}E_{1}^{\text{int}(1)}(\xi_{p}),\;{\hat{\text{D}}}^{\left(1\right)}\in R^{N\times N},$$

which is recast as

2.17 $$\text{F}={\tilde{\text{D}}}^{\left(1\right)}{\tilde{\text{F}}}^{\left(1\right)}+{\text{E}}_{1}^{\text{inv}(1)},\;{\tilde{\text{D}}}^{\left(1\right)}\in R^{N\times (N+1)},$$

where ${\tilde{\text{D}}}^{\left(1\right)}=[{\hat{\text{D}}}^{\left(1\right)}\;\text{I}]$ and ${\tilde{\text{F}}}^{\left(1\right)}={\left[{\text{F}}^{\left(1\right)}\;c_{0}\right]}^{T}$. ${\text{E}}_{1}^{\text{inv}(1)}=\text{I}E_{1}^{\text{int}(1)}(\xi_{p})$.

Equation (2.17) is the equivalent of equation (2.4), which is numerically stable and computationally efficient for the approximation of *f*(*ξ*) from its first derivative.

### (e) Higher-order inverse differential quadrature method-by-inversion

To obtain higher-order iDQM formulae from the DQM counterpart, we revisit equation (2.17) for the first-order derivative, explicitly expressed as

2.18 $$\text{F}={\hat{\text{D}}}^{\left(1\right)}{\text{F}}^{\left(1\right)}+\text{I}c_{0}+\text{I}E_{1}^{\text{int}(1)}(\xi_{p}).$$

Analogously, we can write derivations of first and second derivatives of *f*(*ξ*) in terms of the second and third derivatives, respectively, as:

2.19 $$\begin{array}{rl} & {\text{F}}^{\left(1\right)}={\hat{\text{D}}}^{\left(1\right)}{\text{F}}^{\left(2\right)}+\text{I}c_{1}+\text{I}E_{2}^{\text{int}(1)}(\xi_{p})\\ \;\text{and}\; & {\text{F}}^{\left(2\right)}={\hat{\text{D}}}^{\left(1\right)}{\text{F}}^{\left(3\right)}+\text{I}c_{2}+\text{I}E_{3}^{\text{int}(1)}(\xi_{p}), \end{array}\}$$

where $E_{2}^{\text{int}(1)}$ and $E_{3}^{\text{int}(1)}$ are error estimates implied from equation (2.5) after first-order integration, for *m* = 2, 3, i.e.

2.20 $$\begin{array}{rl} & f^{\left(1\right)}(\xi )=\sum_{i=1}^{N}f_{i}^{\left(2\right)}H^{\left(1\right)}(\xi )+c_{1}+E_{2}^{\text{int}(1)}(\xi )\\ \;\text{and}\; & f^{\left(2\right)}(\xi )=\sum_{i=1}^{N}f_{i}^{\left(3\right)}H^{\left(1\right)}(\xi )+c_{2}+E_{3}^{\text{int}(1)}(\xi ). \end{array}\}$$

By multiplying the first equation of (2.19) by ${\hat{\text{D}}}^{\left(1\right)}$ while adding **I***c*_0_ and $\text{I}E_{1}^{\text{inv}(1)}$ leads to

2.21 $$\underset{\text{F}}{\underbrace{{\hat{\text{D}}}^{\left(1\right)}{\text{F}}^{\left(1\right)}+\text{I}c_{0}+\text{I}E_{1}^{\text{inv}(1)}}}={\hat{\text{D}}}^{\left(1\right)}{\hat{\text{D}}}^{\left(1\right)}{\text{F}}^{\left(2\right)}+{\hat{\text{D}}}^{\left(1\right)}\text{I}c_{1}+\text{I}c_{0}+{\hat{\text{D}}}^{\left(1\right)}\text{I}E_{2}^{\text{int}(1)}(\xi_{p})+\text{I}E_{1}^{\text{inv}(1)},$$

or alternatively as

2.22 $$\text{F}={\hat{\text{D}}}^{\left(2\right)}{\text{F}}^{\left(2\right)}+\xi c_{1}+\text{I}c_{0}+\underset{{\text{E}}_{2}^{\text{inv}(2)}}{\underbrace{\left(\xi E_{2}^{\text{int}(1)}(\xi_{p})+\text{I}E_{1}^{\text{inv}(1)}\right)}},\;{\hat{\text{D}}}^{\left(2\right)}\in R^{N\times N},$$

where ${\hat{\text{D}}}^{\left(2\right)}={\left({\hat{\text{D}}}^{\left(1\right)}\right)}^{2}$ and it can be proved that $\xi ={\hat{\text{D}}}^{\left(1\right)}\text{I}$. ${\text{E}}_{2}^{\text{inv}(2)}$ is the error due to second-order *iDQM-by-inversion* operation. Repeating the same operation as in equations (2.21) and (2.22) twice for the second equation of (2.19), and adding the last three terms in equation (2.22), leads to

2.23 $$\underset{\text{F}}{\underbrace{{\hat{\text{D}}}^{\left(2\right)}{\text{F}}^{\left(2\right)}+\xi c_{1}+\text{I}c_{0}+{\text{E}}_{2}^{\text{inv}(2)}}}={\hat{\text{D}}}^{\left(2\right)}{\hat{\text{D}}}^{\left(1\right)}{\text{F}}^{\left(3\right)}+{\hat{\text{D}}}^{\left(2\right)}\text{I}c_{2}+{\hat{\text{D}}}^{\left(2\right)}\text{I}E_{3}^{\text{int}(1)}(\xi_{p})+\xi c_{1}+\text{I}c_{0}+{\text{E}}_{2}^{\text{inv}(2)},$$

or alternatively as

2.24 $$\text{F}={\hat{\text{D}}}^{\left(3\right)}{\text{F}}^{\left(3\right)}+\frac{1}{2}\xi^{2}c_{2}+\xi c_{1}+\text{I}c_{0}+\underset{{\text{E}}_{3}^{\text{inv}(3)}}{\underbrace{\left(\frac{1}{2}\xi^{2}E_{3}^{\text{int}(1)}(\xi_{p})+{\text{E}}_{2}^{\text{inv}(2)}\right)}},\;{\hat{\text{D}}}^{\left(3\right)}\in R^{N\times N},$$

where ${\hat{\text{D}}}^{\left(3\right)}={\left({\hat{\text{D}}}^{\left(1\right)}\right)}^{3}$ and it can be proved that $(1/2)\xi^{2}={\hat{\text{D}}}^{\left(2\right)}\text{I}$. ${\text{E}}_{3}^{\text{inv}(3)}$ is the error due to third-order *iDQM-by-inversion* operation.

Equations (2.17) and (2.19) represent the second- and third-order equivalent of equation (2.8) from which a vector function **F** can be recovered from its higher derivatives. Accordingly, the general expression for the *m*th-order *iDQM-by-inversion* operation is given as

2.25 $$\text{F}={\hat{\text{D}}}^{\left(m\right)}{\text{F}}^{\left(m\right)}+\frac{1}{(m-1)!}\xi^{m-1}c_{m-1}+,\ldots ,+\xi c_{1}+\text{I}c_{0}+\underset{{\text{E}}_{m}^{\text{inv}(m)}}{\underbrace{\left(\frac{1}{(m-1)!}\xi^{m-1}E_{m}^{\text{int}(1)}(\xi_{p})+{\text{E}}_{m-1}^{\text{inv}(m-1)}\right)}},$$

where ${\text{E}}_{m}^{\text{inv}(m)}$ is the error due to *m*th order *iDQM-by-inversion* operation. The error term in equation (2.25) can be dropped since it is very small compared with **F**. Thus, equation (2.25) is rewritten as

2.26 $$\text{F}\approx {\hat{\text{D}}}^{\left(m\right)}{\text{F}}^{\left(m\right)}+\frac{1}{(m-1)!}\xi^{m-1}c_{m-1}+,\ldots ,+\xi c_{1}+\text{I}c_{0}.\;{\hat{\text{D}}}^{\left(m\right)}\in R^{N\times N}.$$

Note: the subscript and superscript of ${\text{E}}_{m}^{\text{inv}(m)}$ refers, respectively, to the order of function, i.e. *f*^(*m*)^, and the order of inverse operation.

If coordinate transformation is performed for the interval *ξ* ∈ [*a* *b*] to *y* ∈ [0 *L*] while noting from equation (1.3) that *l*_*i*_ (0) = 0, equations (2.9) and (2.26) can be transformed to

2.27 $$\text{F}\approx {\text{H}}^{\left(m\right)}{\text{F}}^{\left(m\right)}+\frac{1}{(m-1)!}{\text{y}}^{m-1}f^{m-1}(0)+,\ldots ,+\text{y}f^{\left(1\right)}(0)+\text{I}f^{\left(0\right)}$$

and

2.28 $$\text{F}\approx {\hat{\text{D}}}^{\left(m\right)}{\text{F}}^{\left(m\right)}+\frac{1}{(m-1)!}{\text{y}}^{m-1}f^{m-1}(0)+,\ldots ,+\text{y}f^{\left(1\right)}(0)+\text{I}f^{\left(0\right)},$$

where **y** = [*y*_1_, *y*_2_, …, *y*_*N*_]^*T*^, $\text{y}\in R^{N\times 1}$.

It should be noted that after transformation, point *ξ*_*p*_ = *y*_*p*_ = 0. Subsequent derivations will be presented in the new coordinates, *y*.

### (f) Proof of ${\text{y}}^{m}=m!{\hat{\text{D}}}^{\left(m\right)}\text{I}$

Suppose an *m*th-order polynomial, *f*(*y*) = *y*^*m*^ can be approximated by DQM as

2.29 $$f(y)=\sum_{i=1}^{N}f(y_{i})l_{i}(y)+E(f),$$

where *l*_*i*_ (*y*), *f*(*y*_*i*_) and *E*(*f*) are described according to equations (1.2)–(1.5). Differentiating equation (2.29) once according to DQM approximation leads to the discretized equation,

2.30 $${\text{F}}^{\left(1\right)}={\text{D}}^{\left(1\right)}\text{F}+{\text{E}}^{\text{dif}(1)}.$$

By evaluating the first derivative of the function *y*^*m*^ analytically, equation (2.30) can be rewritten as

2.31 $$m{\text{y}}^{\left(m-1\right)}={\text{D}}^{\left(1\right)}\text{F}+{\text{E}}^{\text{dif}(1)},$$

which is further simplified (via inversion) after rearrangement in line with procedures in §d as

2.32 $$\text{F}=m{\bar{\text{D}}}^{\left(1\right)}{\text{y}}^{\left(m-1\right)}+\text{I}{\bar{c}}_{0}.$$

In line with equation (2.13), if a point exists in the closed interval *y* ∈ [0 *L*] where *f*(0) equals the constant *c*_0_, then ${\bar{c}}_{0}$ can be derived in terms of *c*_0_ as

2.33 $${\bar{c}}_{0}=c_{0}-m{\left[{\bar{\text{D}}}^{\left(1\right)}\right]}_{1}{\text{y}}^{\left(m-1\right)}.$$

Note that equation (2.33) is exact since integration of *f*(*y*) = *y*^*m*^ is exact. Additionally, *p* = 1 since *y*_*p*_ = 0 is the first point in the interval *y* ∈ [0 *L*]. Then, equation (2.33) can be substituted into equation (2.32) to give

2.34 $$\text{F}=\underset{{\hat{\text{D}}}^{\left(1\right)}}{\underbrace{\left({\bar{\text{D}}}^{\left(1\right)}-\text{I}{\left[{\bar{\text{D}}}^{\left(1\right)}\right]}_{1}\right)}}m{\text{y}}^{\left(m-1\right)}+\text{I}c_{0},$$

which is now simplified as

2.35 $$\text{F}=m{\hat{\text{D}}}^{\left(1\right)}{\text{y}}^{\left(m-1\right)}+\text{I}c_{0},$$

where *c*_0_ is the intercept of *f*(*y*). Since *f*(*y*) = *y*^*m*^, such that the intercept *c*_0_ = 0, then equation (2.35) becomes

2.36 $$\text{F}={\text{y}}^{m}=m{\hat{\text{D}}}^{\left(1\right)}{\text{y}}^{\left(m-1\right)},\;\text{for}\;m=1,2,\ldots$$

By considering that $y={\hat{\text{D}}}^{\left(1\right)}\text{I}$ from equation (2.36), we can recast equation (2.36) after successive expansion as

2.37 $${\text{y}}^{m}=m!{\hat{\text{D}}}^{\left(m\right)}\text{I},$$

where ${\hat{\text{D}}}^{\left(m\right)}={\left({\hat{\text{D}}}^{\left(1\right)}\right)}^{m}$. This completes the proof.

## 3. Inverse differential quadrature method error

It is important to formulate an iDQM error estimate to describe the performance of iDQM in terms of numerical accuracy and numerical stability. Since *iDQM-by-inversion* is adopted in this work to circumvent computational issues arising from analytical or numerical integration, there is also a need to quantify the error estimate due to DQM inversion in order to assess the approximation quality of the proposed *iDQM-by-inversion*. Moreover, as the DQM coefficient matrix is used to compute iDQM weighting factors, a comparison of discrepancy between DQM and *iDQM-by-inversion* error estimates is necessary to confirm improvements and drawbacks of the proposed method.

### (a) Error formulation of inverse differential quadrature method-by-integration

Consider a continuously differentiable function up to *m*th degree, *f*(*y*), where *m* is very high,

3.1 $$f(y)=y^{m},\;\text{for}\;m>1.$$

Approximation of the first-order derivative of *f*, *f*^(1)^, according to equation (2.1) gives

3.2 $$f_{N}^{\left(1\right)}(y)\approx \sum_{i=1}^{N}f_{i}^{\left(1\right)}l_{i}(y),\;\text{s.t.}\;N<<m.$$

To determine the approximation error, a function, *F*(*z*), is defined such that

3.3 $$F(z)=f^{\left(1\right)}(z)-f_{N}^{\left(1\right)}(z)-bM_{N}(z),$$

where *M*_*N*_ (*y*) is a *N* degree polynomial defined in equation (1.3), and *b* is a constant. It is noted that when *z* = *y*_1_, *y*_2_, …, *y*_*N*_, *F*(*z*) = 0. So, by setting *F*(*y*) = 0, the approximation error is estimated as

3.4 $$E_{1}(y)=f^{\left(1\right)}(y)-f_{N}^{\left(1\right)}(y)=bM_{N}(y).$$

Considering equation (3.3), it is noted that *F*(*z*) has *N* roots, *y*_1_, *y*_2_, …, *y*_*N*_, in the domain *y* ∈ [0 *L*]. Applying Rolle’s theorem [51] repeatedly leads to the *N*th-order derivative of *F*(*z*), *F*^*N*^ (*z*), which has at least one root, *μ*, between *y*_1_ and *y*_*N*_ leading to

3.5 $$F^{N}(\mu )=0,$$

while noting that $f_{N}^{\left(1\right)}(z)$ is a polynomial of (*N* − 1) degree. In this instance,

3.6 $$b=\frac{f^{N+1}(\mu )}{N!}.$$

Substituting equation (3.6) into equation (3.4) gives

3.7 $$E_{1}(y)=\frac{f^{N+1}(\mu )}{N!}M_{N}(y).$$

In general, *μ* is a function of *y*.

It is noted in equation (3.7) that *E*_1_ (*y*_*i*_) = 0, since *M*_*N*_ (*y*_*i*_) = 0. In line with equation (3.7), let us denote the maximum error of *f*^(1)^ approximation for a fixed order of *y* as $E_{1_{\text{max}}}$, which is expressed as

3.8 $$E_{1_{\text{max}}}(y)=\frac{{\hat{K}}_{1}M_{N}(y)}{N!},$$

where ${\hat{K}}_{1}=|f^{N+1}(\mu ){|}_{\text{max}}$. Now, the maximum error estimate of the original function, *f*(*y*), recovered via applying *iDQM-by-integration* to equation (3.7) is obtained thus

3.9 $$E_{1_{\text{max}}}^{\text{int}(1)}(y)=\frac{{\hat{K}}_{1}M_{N}^{\text{int}(1)}(y)}{N!},$$

where $M_{N}^{\text{int}(1)}$ and $E_{1_{\text{max}}}^{\text{int}(1)}$ are the first-order integrals of *M*_*N*_ (*y*) and $E_{1_{\text{max}}}$, respectively. Since ${\hat{K}}_{1}$ and *N* are constants, it can be stated that the total error arising from numerical integration is constrained by $M_{N}^{\text{int}(1)}$. To have a clearer understanding of the implication of equation (3.9), consider a specific case of Chebyshev grid distribution, where *y*_*i*_ are the roots of the Chebyshev polynomial, *T*_*k*_ (*y*), of order *k*. In this context, *y*_*i*_ can be expressed as

3.10 $$y_{i}=\cos\theta_{i},\;N\theta_{i}=i\pi \;\text{for}\;i=0,1,\ldots ,N.$$

In connection with equation (3.10), the polynomial, *M*_*N*_ (*y*), can be expressed in terms of *T*_*k*_ (*y*) as (see [23])

3.11 $$M_{N}(y)=(1-y^{2})T_{N}^{\left(1\right)}(y),$$

where $T_{N}^{\left(1\right)}(y)$ is the first-order derivative of *T*_*N*_ (*y*). Setting *y* = *cosθ* and *T*_*N*_ (*y*) = *cos Nθ* in equation (3.11) leads to,

3.12 $$M_{N}(y)=M_{N}(\theta )=N\sin\theta \;\sin N\theta .$$

The first-order integral of *M*_*N*_ (*y*) in equation (3.12) is expressed as

3.13 $$M_{N}^{\text{int}(1)}(y)=\left\{ \begin{array}{ll} 0 & \text{for}\;N=0\\ \frac{N\cos N\theta }{2N}-\frac{N\cos(N\theta -2\theta )}{4N-8}-\frac{N\cos(N\theta +2\theta )}{4N+8} & \text{for}\;N\neq 0,2\\ -\sin^{4}\theta & \text{for}\;N=2. \end{array}\right.$$

Subject to equation (3.10), equation (3.13) can be recast in terms of *y*_*i*_ at the grid points as:

3.14 $$M_{N}^{\text{int}(1)}(y_{i})=\left\{ \begin{array}{ll} 0 & \text{for}\;N=0\\ N{\left(-1\right)}^{i}(\frac{1}{2N}-\frac{2y_{i}^{2}-1}{4N-8}-\frac{2y_{i}^{2}-1}{4N+8}) & \text{for}\;N\neq 0,2\\ -{\left(1-y_{i}^{2}\right)}^{2} & \text{for}\;N=2. \end{array}\right.$$

As $M_{N}^{\text{int}(1)}(y_{i})$ varies in *N* by order *O*(*N*/*N*) in equation (3.14), it can be deduced that for a fixed order of *y*, the accuracy of iDQM operations is not substantially affected after first-order integration as *N* increases, since *O*(*N*/*N*) is mutually compensating (i.e. varies directly and inversely equally in order of *N*).

The error for recovering *f* from its second-order derivative, *f*^(2)^, can be obtained by redefining equation (3.3) as

3.15 $$F(z)=f^{\left(2\right)}(z)-f_{N}^{\left(2\right)}(z)-bM_{N}(z).$$

Then, repeating the procedures of equations (3.4)–(3.8) leads to the maximum error for *f* approximation from *f*^(2)^ as

3.16 $$E_{2_{\text{max}}}(y)=\frac{{\hat{K}}_{2}M_{N}(y)}{N!},$$

where ${\hat{K}}_{2}=|f^{N+2}(\mu ){|}_{\text{max}}$. The maximum iDQM error estimate of the original function, *f*(*y*), recovered via second-order *iDQM-by-integration* gives

3.17 $$E_{2_{\text{max}}}^{\text{int}(2)}(y)=\frac{{\hat{K}}_{2}M_{N}^{\text{int}(2)}(y)}{N!}.$$

Adopting a Chebyshev grid representation as applied in equations (3.10)–(3.14) results in

3.18 $$M_{N}^{\text{int}(2)}(y_{i})={\left(-1\right)}^{i}\frac{N}{8}\left[(4y_{i}^{3}-3y_{i})\left[\frac{1}{{\left(N-3\right)}^{2}}-\frac{1}{{\left(N+3\right)}^{2}}\right]+3y_{i}\left[\frac{1}{{\left(N+1\right)}^{2}}-\frac{1}{{\left(N-1\right)}^{2}}\right]\right].$$

A similar approach can be used to recover the maximum iDQM approximation error of *f*(*y*) from its third-order derivative, *f*^(3)^, as,

3.19 $$E_{3_{\text{max}}}^{\text{int}(3)}(y)=\frac{{\hat{K}}_{3}M_{N}^{\text{int}(3)}(y)}{N!},$$

where ${\hat{K}}_{3}=|f^{N+3}(\mu ){|}_{\text{max}}$, and $M_{N}^{\text{int}(3)}(y)$ in the context of Chebyshev grid is given by

3.20 $$\begin{array}{rl} M_{N}^{\text{int}(3)}(y_{i}) & ={\left(-1\right)}^{i}\frac{N}{16}[-(8y_{i}^{4}-8y_{i}^{2}+1)\left[\frac{1}{{\left(N-4\right)}^{3}}+\frac{1}{{\left(N+4\right)}^{3}}\right]\\ & \;+4(2y_{i}^{2}-1)\left[\frac{1}{{\left(N-2\right)}^{3}}+\frac{1}{{\left(N+2\right)}^{3}}\right]-\frac{6}{N^{3}}]. \end{array}$$

Equations (3.18) and (3.20) show that $M_{N}^{\text{int}(2)}$ varies in *N* by order *O*(*N*/*N*^2^) while $M_{N}^{\text{int}(3)}$ varies in *N* by order *O*(*N*/*N*^3^). This observation illustrates that, for high-order approximation due to *iDQM-by-integration*, the approximation error is scaled by a function which varies inversely in multiple orders of *N*. Therefore, subject to a fixed order of *y*, high-order *iDQM-by-integration* operation is potentially stable numerically as *N* increases.

### (b) Error of inverse differential quadrature method-by-inversion

According to equation (2.17), the approximation error for first-order *iDQM-by-inversion* reads

3.21 $$E_{1}^{\text{inv}(1)}=E_{1}^{\text{int}(1)}(0).$$

Noting equation (3.9), the maximum approximation error for first-order *iDQM-by-inversion* becomes

3.22 $$E_{1_{\text{max}}}^{\text{inv}(1)}=\frac{{\hat{K}}_{1}M_{N}^{\text{int}(1)}(0)}{N!},$$

where $M_{N}^{\text{int}(1)}(0)$ given in the context of Chebyshev polynomials reads

3.23 $$M_{N}^{\text{int}(1)}(0)=\left\{ \begin{array}{ll} 0 & \text{ for }N=0\\ N{\left(-1\right)}^{i}(\frac{1}{2N}+\frac{1}{4N-8}+\frac{1}{4N+8}) & \text{ for }N\neq 0,2\\ -1 & \text{for }N=2. \end{array}\right.$$

As in equation (3.14), $M_{N}^{\text{int}(1)}(0)$ varies in *N* by order *O*(*N*/*N*) implying that, as *N* increases, the contribution of $M_{N}^{\text{int}(1)}(0)$ to the total approximation error in equation (3.22) is expected to be minimal. In the same vein, the second-order approximation error according to *iDQM-by-inversion* is obtained according to equation (2.22),

3.24 $$E_{2}^{\text{inv}(2)}(y)=yE_{2}^{\text{int}(1)}(0)+E_{1}^{\text{int}(1)}(0).$$

By substituting for $E_{2}^{\text{int}(1)}(0)$ and $E_{1}^{\text{int}(1)}(0)$ in equation (3.24) using equations (3.9) and (3.17), the maximum approximation error due to second-order *iDQM-by-inversion* operation gives

3.25 $$E_{2}^{\text{inv}(2)}=y\frac{{\hat{K}}_{2}M_{N}^{\text{int}(1)}(0)}{N!}+\frac{{\hat{K}}_{1}M_{N}^{\text{int}(1)}(0)}{N!}.$$

To establish a relationship between ${\hat{K}}_{2}$ and${\hat{K}}_{1}$, consider the *N*th derivative of *f*(*y*) expressed as

3.26 $$f^{N}(y)=\frac{m!}{(m-N)!}y^{m-N}.$$

Furthermore, a recursive relation can be established between a derivative and its lower-order derivative as

3.27 $$f^{N+1}(y)=\frac{m!}{(m-N-1)!}y^{m-N-1}=\frac{\left(m-N\right)}{y}f^{N}(y)$$

and

3.28 $$f^{N+2}(y)=\frac{m!}{(m-N-2)!}y^{m-N-2}=\frac{\left(m-N-2\right)}{y}f^{N+1}(y).$$

Noting this relation, it can be established that

3.29 $$f^{N+2}(\mu )=\frac{\left(m-N-1\right)}{\mu }f^{N+1}(\mu )$$

and

3.30 $${\hat{K}}_{2}=\frac{\left(m-N-1\right)}{\mu (y)}{\hat{K}}_{1}.$$

Substituting equation (3.30) into equation (3.25), the approximation of the second-order iDQM inversion gives

3.31 $$E_{2_{\text{max}}}^{\text{inv}(2)}=\left(\frac{y(m-N-1)}{\mu (y)}+1\right)\frac{{\hat{K}}_{1}M_{N}^{\text{int}(1)}(0)}{N!},$$

which is further simplified to

3.32 $$E_{2_{\text{max}}}^{\text{inv}(2)}=\left(\frac{y(m-N-1)}{\mu (y)}+1\right)E_{1_{\text{max}}}^{\text{inv}(1)}.$$

Applying similar procedures as in equations (3.24)–(3.32) leads to the approximation error for the third-order DQM inversion:

3.33 $$E_{3_{\text{max}}}^{\text{inv}(3)}=\left(\frac{y^{2}(m-N-3)(m-N-2)}{\mu^{2}(y)}+\frac{y(m-N-1)}{\mu (y)}+1\right)E_{1_{\text{max}}}^{\text{inv}(1)}.$$

According to equations (3.31) and (3.33), high-order approximation by *iDQM-by-inversion* incurs progressive error, which increases successively by order *N*.

### (c) Comparison of inverse differential quadrature method error with differential quadrature method error

As noted in Shu [23], the attributable error as a result of first-order DQM approximation is given by

3.34 $$E(y)=\frac{f^{N}(\mu )}{N!}M_{N}(y),$$

where *μ* is a function of *y*. Considering equation (3.8), the maximum error of function approximation by DQM is analogously expressed as

3.35 $$E_{\text{max}}(y)=\frac{K_{1}M_{N}(y)}{N!},$$

where *K*_1_ = |*f*^*N*^ (*μ*)|_max_. By differentiating equation (3.35) to obtain the error due to first-order numerical differentiation, we get

3.36 $$E_{\text{max}}^{\text{dif}(1)}(y)=\frac{K_{1}M_{N}^{\text{dif}(1)}(y)}{N!},$$

where $M_{N}^{\text{dif}(1)}(y)$ and $E_{\text{max}}^{\text{dif}(1)}$ are the first derivatives of *M*_*N*_ (*y*) and *E*_max_, respectively. According to equation (3.36), the error arising from first-order DQM differentiation is constrained by $M_{N}^{\text{dif}(1)}(y)$, since *K*_1_ and *N* are constants. Adopting Chebyshev grid representation, considering equations (3.11)–(3.12), the first derivative of *M*_*N*_ (*y*) is expressed as

3.37 $$M_{N}^{\text{dif}(1)}(y)=-\frac{N\sin N\theta \;\cos\theta +N^{2}\cos N\theta }{\sin\theta }.$$

Substituting for *y*_*i*_ at the Chebyshev grid points while noting equation (3.10) results in

3.38 $$M_{N}^{\text{dif}(1)}(y_{i})=\left\{ \begin{array}{ll} {\left(-1\right)}^{i+1}N^{2} & \text{for }i\neq 0,N\\ -2N^{2} & \text{for }i=0,N. \end{array}\right.$$

In the same vein, the maximum error due to second-order DQM approximation is given as

3.39 $$E_{\text{max}}^{\text{dif}(2)}(y)=\frac{K_{1}M_{N}^{\text{dif}(2)}(y)}{N!}.$$

Evaluating $M_{N}^{\text{dif}(2)}(y)$ in the context of Chebyshev polynomials leads to

3.40 $$\begin{array}{rl} M_{N}^{\text{dif}(2)}(y) & =\frac{2N^{2}\cos N\theta \cos\theta -N\sin N\theta \sin\theta -N^{3}\sin N\theta \sin\theta }{\sin^{2}\theta }\\ & \;-\frac{N\sin N\theta \cos^{2}\theta -N^{2}\cos N\theta \sin\theta \cos\theta }{\sin^{3}\theta }. \end{array}$$

Substituting for *y*_*i*_ in equation (3.40) at the Chebyshev grid points using equation (3.10) gives

3.41 $$M_{N}^{\text{dif}(2)}(y_{i})=\left\{ \begin{array}{ll} -\frac{2}{3}N^{2}(1+2N^{2}) & \text{for }i=0\\ {\left(-1\right)}^{i}N^{2}\frac{y_{i}}{1-y_{i}^{2}} & \text{for }i\neq 0,N\\ {\left(-1\right)}^{i}\frac{2}{3}N^{2}(1+2N^{2}) & \text{for }i=N. \end{array}\right.$$

According to equation (3.38), first-order DQM approximation is constrained by $M_{N}^{\text{dif}(1)}$ which varies in *N* by order *O*(*N*^2^). For this reason, $M_{N}^{\text{dif}(1)}$ magnifies the total DQM approximation error subject to first-order numerical differentiation. Second-order DQM approximation gives rise to a total approximation error constrained by $M_{N}^{\text{dif}(2)}$ , which varies in *N* by order *O*(*N*^4^) leading to further magnification of the total error as the order of numerical differentiation increases to 2. In general, for a fixed order of numerical differentiation or numerical integration, both DQM and iDQM total errors decrease as *N* increases as they vary each in *N* by order *O*(1/*N*^*N*^). However, the contributions of $M_{N}^{\text{dif}(1)}$ and $M_{N}^{\text{int}(1)}$ to the total errors subject to first-order numerical differentiation and numerical integration, respectively, affect the overall accuracy and numerical stability of the approximations. Comparing *iDQM-by-integration* and DQM error representation in this regard, it can be deduced that, for a fixed order of y, the resulting error from first-order *iDQM-by-integration* is less than the first-order DQM approximation, as the total error magnification on account of the order of $M_{N}^{\text{dif}(1)}$ and $M_{N}^{\text{int}(1)}$, respectively, in *N* is higher for DQM than iDQM approximation. This observation is also true for high-order *iDQM-by-integration* operation since, given a fixed order of *y*, the contribution of $M_{N}^{\text{int}(1)}$ to the total error is less than the contribution of $M_{N}^{\text{dif}(1)}$ to the total high-order differentiation error of DQM operations. Although *iDQM-by-inversion* operation progressively magnifies the approximation error by order *N*, the rate of increase in the order of *N* for every successive inversion by iDQM operation is less than the rate of increase in the order of *N* for every successive differentiation by DQM. Therefore, iDQM operations in general potentially demonstrate superior numerical stability than DQM operations.

## 4. Numerical results and discussions

In this section, we present some illustrations of the numerical accuracy and numerical stability of the proposed iDQM for functional approximation as well as solution of ordinary differential equations (ODEs) and partial differential equations (PDEs) representing linear and nonlinear systems. We further demonstrate how different schemes of iDQM can be implemented using several examples. The results obtained are then benchmarked with DQM and exact solutions to evaluate the performance of iDQM solutions. To show the robustness of iDQM solutions, numerical analyses based on Lagrange basis polynomials on a non-uniform Chebyshev grid structure are performed and the errors computed. In addition, a comprehensive error analysis to examine the performance of iDQM schemes in terms of convergence and error propagation is performed using a fourth-order boundary value problem (BVP). The results are benchmarked against DQM and exact solutions to evaluate the gains of iDQM approximations.

Note: all examples performed in this work are implemented using *iDQM-by-inversion* since it proves more computationally efficient than *iDQM-by-integration*.

### (a) Approximation of function and its higher derivatives

Consider the function,

4.1 $$f=0.02(12+3y-3.5y^{2}+7.2y^{3})\;(1+cos4\pi y)\;(1+0.8sin3\pi y),\;\forall \;y\in [0\;\;1].$$

**Figure 1.**
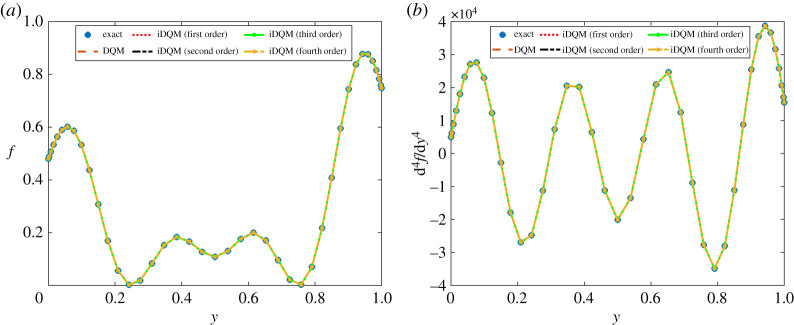
iDQM results implemented for approximation of (*a*) function and (*b*) fourth derivative. (Online version in colour.)

Function *f* and its derivates up to fourth order are approximated by iDQM schemes of different orders. The results shown in figure 1 prove the accuracy of iDQM approximation as they agree satisfactorily with DQM and exact solutions. The measured relative error, *ϵ*, between iDQM and DQM estimates and exact solution is computed using the relation,

4.2 $$\epsilon =\frac{||\text{f}-{\text{f}}_{\text{approx}}|{|}_{2}}{||\text{f}|{|}_{2}},$$

where **f** is a vector function consisting of exact values of *f* evaluated at each point in the domain while **f**_approx_ represents a vector function of approximate estimates of *f* at each point in the domain. All error plots presented in subsequent examples are computed using equation (4.2).

According to table 1, iDQM estimates of *f* in equation (4.1) and its derivatives prove more accurate than DQM estimates. This observation is more evident for second- and third-order iDQM schemes in which the total error is less perturbed by numerical differentiation by DQM (to obtain high-order derivatives) or numerical integration by iDQM (to obtain low-order derivatives). Depending on the order of iDQM, the accuracy of functional approximations may fluctuate between low-order derivative approximation and low-order integral approximation, which is beneficial compared with DQM estimates, which accumulate error subject to successive high-order numerical differentiation. Apart from this, the rate of decline in numerical accuracy for DQM estimates is higher than for iDQM estimates, which is a good indication of numerical stability in favour of iDQM. This aspect is discussed further in §4.g.

**Table 1.** Maximum absolute error (relative error) of iDQM and DQM estimates for functional approximation.

	41 points				
	DQM	first-order iDQM	second-order iDQM	third-order iDQM	fourth-order iDQM
*f*	0	10^−15^(10^−15^)	10^−14^(10^−14^)	10^−14^(10^−14^)	10^−12^(10^−12^)
*f*^(1)^	10^−13^(10^−15^)	0	10^−14^(10^−14^)	10^−13^(10^−14^)	10^−12^(10^−13^)
*f*^(2)^	10^−11^(10^−13^)	10^−12^(10^−15^)	0	10^−13^(10^−15^)	10^−11^(10^−13^)
*f*^(3)^	10^−8^(10^−12^)	10^−9^(10^−14^)	10^−11^(10^−15^)	0	10^−11^(10^−14^)
*f*^(4)^	10^−5^(10^−11^)	10^−7^(10^−12^)	10^−8^(10^−14^)	10^−10^(10^−15^)	0

### (b) Solution schemes for systems of differential equations by the inverse differential

quadrature method

By approximation of higher derivatives instead of the original function, the proposed iDQM formulation presents a unique opportunity to tune the order of a system, which aids in control of numerical accuracy and numerical stability of the system. In this context, two concepts are proposed in the following subsections.

#### (i) Mixed inverse differential quadrature method

This scheme involves combination of DQM and iDQM in a manner that ensures application of iDQM for approximation of intermediate derivative(s), which is lower than the highest derivative in a system, such that lower-order functions can be obtained via iDQM integration while higher-order functions are obtained via DQM differentiation. For example, a fourth-order system of equations can be represented by approximation of the second-order derivative in a MiDQM scheme, in which case the first-order derivative and original function are obtained by numerical integration while third- and fourth-order derivatives are obtained by numerical differentiation. This strategy leads to reduction in the DQM order required for the system solution and, by implication, reduction of the order of the DQM approximation error. This approach is highly promising, and therefore noteworthy, in that it allows tuning of the numerical accuracy of DQM to achieve improved solution. A demonstration of the implementation of this approach is presented for one dimension and two dimensions in appendix A.

#### (ii) Full inverse differential quadrature method

In contradistinction to its DQM counterpart, FiDQM presents an opportunity to approximate the highest derivative in a system and then apply equation (2.28) to retrieve lower derivatives via iDQM operation. As established in the previous section, given a geometry, the error accrued by integrating a high-order function to get low-order estimates is quite stable numerically compared with differentiating low-order functions to get high-order estimates. As a result, depending on properties such as geometric specifications, boundary conditions or order of a system, high numerical accuracy can be achieved potentially by FiDQM schemes compared with DQM. A demonstration of the implementation of this approach is presented for one dimension and two dimensions in appendix A.

### (c) Numerical solution of Euler cantilever beam (ODE)

**Figure 2.**
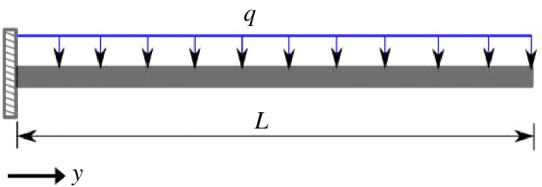
Euler cantilever beam under uniform *q* load. *L* = 10 m, *b* = 0.01 m, *h* = 0.01 m, *E* = 9.05 GPa and q=−1 N. (Online version in colour.)

Consider a Euler cantilever beam under a uniformly distributed load, *q* (figure 2), the governing equation together with the boundary conditions for the beam deflection, *w*, reads

4.3 $$\begin{array}{rl} & EI\frac{{\text{d}}^{4}w}{\text{d}y^{4}}=q,\;\forall \;y\;\in \;[0\;\;L],L=10\;\text{m}\\ \;\text{and}\; & w(y=0)=0,\;\frac{\text{d}w}{\text{d}y}{|}_{y=0}=0,\;\frac{{\text{d}}^{2}w}{\text{d}y^{2}}{|}_{y=L}=0,\;\frac{{\text{d}}^{3}w}{\text{d}y^{3}}{|}_{y=L}=0, \end{array}\}$$

where *E* is Young’s modulus of the beam and *I* is the second moment of area of the beam’s cross-section. According to [52], the exact solution of the beam deflection is given by

4.4 $$w(y)=\frac{qy^{2}(6L^{2}-4Ly+y^{2})}{24EI}.$$

Given the iDQM discretization scheme described in appendix A, it is noted that the equations arising from iDQM constitute an underdetermined system because the number of unknowns exceed the number of equations. In this context, a pseudoinverse procedure based on truncated singular value decomposition described in [53] is adopted in this work to solve the systems of equations.

**Figure 3.**
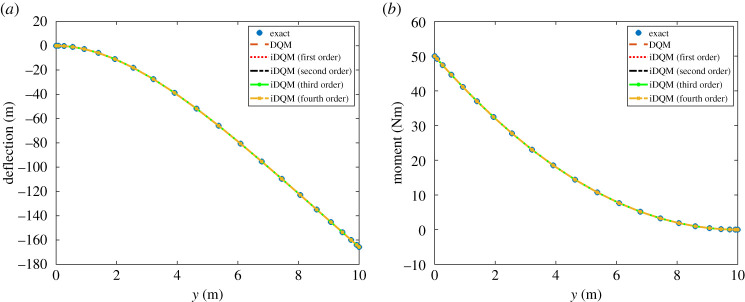
iDQM results for Euler beam (*a*) deflection and (*b*) moment. (Online version in colour.)

According to figure 3, all iDQM estimates of the deflection and moment agree well with exact and DQM solutions showing the accuracy of iDQM solutions. In addition, according to table 2, the relative errors due to iDQM estimates computed for beam deflection, moment and shear force show significant improvement over DQM estimates for the same number of points. This remarkable improvement shows the great potential of iDQM schemes for numerical solution of ordinary differential equations.

**Table 2.** Maximum absolute error (relative error) of iDQM and DQM estimates for Euler cantilevered beam.

	five points				
	DQM	first-order iDQM	second-order iDQM	third-order iDQM	fourth-order iDQM
deflection	10^−12^(10^−14^)	10^−13^(10^−15^)	10^−13^(10^−16^)	10^−13^(10^−16^)	10^−13^(10^−16^)
moment	10^−12^(10^−14^)	10^−13^(10^−14^)	10^−14^(10^−15^)	10^−14^(10^−15^)	10^−14^(10^−16^)
shear force	10^−13^(10^−14^)	10^−14^(10^−15^)	10^−14^(10^−15^)	10^−14^(10^−15^)	10^−14^(10^−16^)

### (d) Nonlinear steady-state solution of heat conduction in slab with temperature-dependent conductivity (ODE)

The problem involves a steady-state heat conduction in a slab with temperature-dependent thermal conductivity in which the non-dimensional form of the temperature (*θ*) governing equation is expressed as

4.5 $$(1+\theta )\frac{{\text{d}}^{2}\theta }{\text{d}\psi^{2}}+{\left(\frac{d\theta }{d\psi }\right)}^{2}=0,\;0\leq \psi \leq 1,\;\theta (\psi =0)=0,\;\theta (\psi =1)=1.$$

The exact solution of the problem is given in [54] as

4.6 $$\theta =-1+\sqrt{1+3\psi }.$$

**Figure 4.**
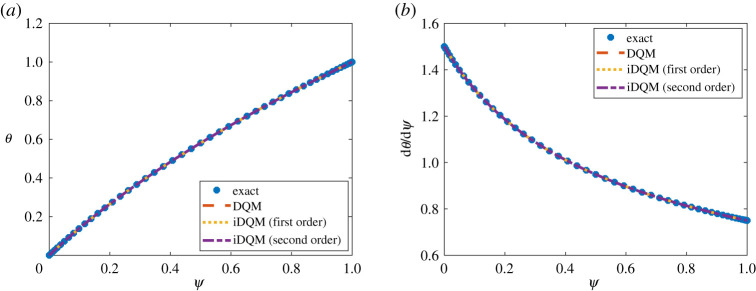
Nonlinear steady-state solution of heat conduction for (*a*) *θ* and (*b*) ∂*θ*/∂*ψ*. (Online version in colour.)

The solution of the nonlinear system, i.e. equation (4.5), is obtained based on Newton–Raphson optimization after iDQM discretization. iDQM results prove accurate with respect to the exact solution and DQM estimates as temperature profile of the slab as well as its first-order derivative match satisfactorily (figure 4). As expected, the error estimates reported in table 3 demonstrate that, although the accuracy of iDQM and DQM estimates of the temperature variable compares equally, iDQM estimates of higher-order derivatives of the temperature variable proves more accurate than DQM estimate, suggesting that error propagation during iDQM operations is less than for DQM operations.

**Table 3.** Maximum absolute error (relative error) of iDQM and DQM estimates for temperature and its derivatives.

	30 points		
	DQM	first-order iDQM	second-order iDQM
*θ*	10^−15^(10^−15^)	10^−16^(10^−16^)	10^−15^(10^−16^)
$\frac{\partial \theta }{\partial \psi }$	10^−13^(10^−14^)	10^−15^(10^−15^)	10^−15^(10^−15^)
$\frac{\partial^{2}\theta }{\partial \psi^{2}}$	10^−11^(10^−12^)	10^−13^(10^−13^)	10^−14^(10^−15^)

### (e) Solution of convection-diffusion equation (PDE)

Consider a steady-state convection-diffusion equation with boundary conditions as follows,

4.7 $$\begin{array}{rl} & \frac{\partial^{2}u}{\partial x^{2}}+\frac{\partial^{2}u}{\partial y^{2}}-P_{e}\frac{\partial u}{\partial x}=0,\;0\leq x,y\leq 1,\\ & u(x,0)=u(x,1)=0,\;0\leq x\leq 1,\;u(0,y)=\sin(\pi y),\;u(1,y)=2\sin(\pi y),\;0\leq y\leq 1, \end{array}$$

where *P*_*e*_ is the Peclet number. The exact solution for the given partial differential equation is given in [55] as

4.8 $$u(x,y)={\text{e}}^{\left(P_{e}x/2\right)}\;\sin(\pi y)\left(\frac{2{\text{e}}^{\left((-P_{e})/2\right)}\;\sinh(\sigma x)+\sinh(\sigma (1-x))}{\sinh(\sigma )}\right),$$

where $\sigma =\sqrt{\pi^{2}+P_{e}^{2}/4}$.

**Figure 5.**
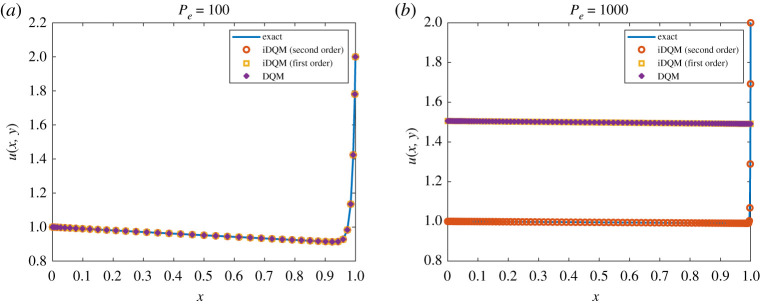
DQM and iDQM *u*(*x*, *y*) estimates at *y* = *b*/2 for *P*_*e*_ (*a*) 100 and (*b*) 1000. (Online version in colour.)

**Figure 6.**
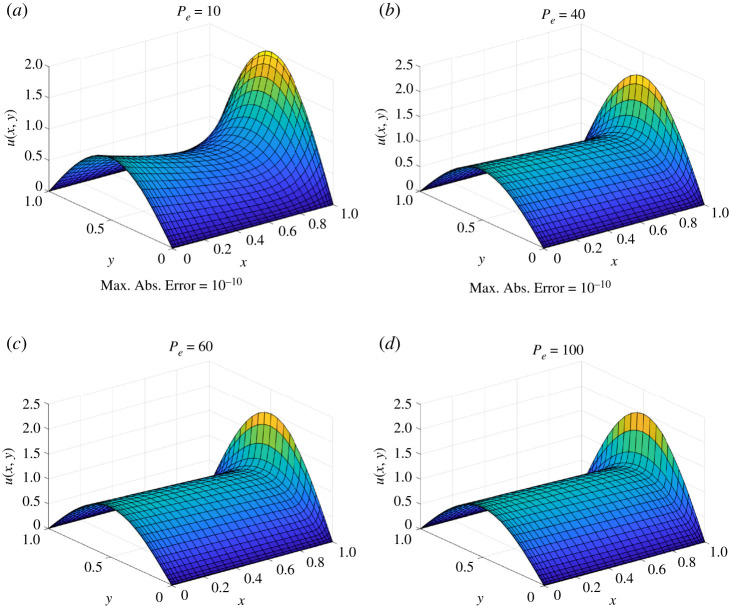
*U*(*x*, *y*) estimate for convection-diffusion equation by iDQM (second order) (31×31 grid). (*a*) Max. Abs. Error=10^−10^, (*b*) Max. Abs. Error=10^−10^, (*c*) Max. Abs. Error=10^−7^ and (*d*) Max. Abs. Error=10^−5^. (Online version in colour.)

According to figure 5, DQM and iDQM values converge to an exact solution of the PDE in equation (4.7) for a 41 × 41 grid, whereas in figure 5, only second-order iDQM converges to the exact solution for a 101 × 101 grid. It is quite evident from table 4 that the accuracy of the numerical values has a strong dependence on the Peclet number. For the case of *Pe* = 1000, DQM and first-order iDQM do not converge to the exact solution for the 101 × 101 grid and further grid refinement fails to improve the solution. According to the findings in [38], an upwind scheme is necessary to obtain accurate solutions for a high Peclet number. Nonetheless, second-order iDQM furnishes an accurate solution for *Pe* = 1000 without an upwind scheme, showing the accuracy of the proposed iDQM. Figure 6 demonstrates the agreement of second-order iDQM with an exact solution over the entire domain.

**Table 4.** Maximum absolute error (relative error) of iDQM and DQM *u*(*x*, *y*) estimates.

grid	first-order iDQM	second-order iDQM	DQM
*P*_*e*_ = 10			
11 × 11	10^−2^(10^−3^)	10^−5^(10^−5^)	10^−2^(10^−2^)
15 × 15	10^−5^(10^−6^)	10^−8^(10^−8^)	10^−6^(10^−6^)
21 × 21	10^−6^(10^−7^)	10^−10^(10^−11^)	10^−11^(10^−12^)
*P*_*e*_ = 40			
11 × 11	10^−1^(10^−1^)	10^−2^(10^−2^)	10^−1^(10^−1^)
21 × 21	10^−2^(10^−2^)	10^−5^(10^−6^)	10^−2^(10^−2^)
31 × 31	10^−7^(10^−7^)	10^−10^(10^−10^)	10^−8^(10^−9^)
*P*_*e*_ = 100			
21 × 21	10^−1^(10^−1^)	10^−3^(10^−3^)	10^−1^(10^−1^)
31 × 31	10^−1^(10^−1^)	10^−5^(10^−5^)	10^−1^(10^−1^)
41 × 41	10^−5^(10^−5^)	10^−8^(10^−8^)	10^−5^(10^−5^)
*P*_*e*_ = 1000			
101 × 101	—	10^−5^(10^−5^)	—

### (f) Solution of simply supported isotropic plate under sinusoidally distributed load (PDE)

We consider a thin isotropic plate with dimensions *a* and *b* in *x* and *y* coordinates, respectively, simply supported on all the edges and under sinusoidally distributed load. The governing differential equation and associated boundary conditions are given as follows,

4.9  ∂4w∂x4+2∂4w∂x2∂y2+∂4w∂y4=qD,0≤x,y≤a,b w(x,0)=w(x,a)=w(0,y)=w(b,y)=0and∂2w∂x2(0,y)=∂2w∂x2(a,y)=∂2w∂y2(x,0)=∂2w∂y2(x,b)=0,}

where *w* is the transverse deflection of the mid-plane of the plate under loading *q*(*x*, *y*) = *q*_0_sin (*πx*/*a*)sin (*πy*/*b*), *q*_0_ is the amplitude of the sinusoidally distributed load, *D* is the flexural stiffness of the plate given as *D* = (*Eh*^3^)/(12(1 − *ν*^2^)), *E* is Young’s modulus, *ν* is Poisson’s ratio and *h* is the thickness of the plate. Material and geometric properties of the plate are given by *E* = 200 GPa, *ν* = 0.3, *a* = *b* = 1 m, *h* = 0.01 m and *q*_0_ = 1 Pa.

The Navier’s closed-form solution is simply given by,

4.10 $$w(x,y)=\frac{q_{0}}{\pi^{4}D{\left(\frac{1}{a^{2}}+\frac{1}{b^{2}}\right)}^{2}}\sin\left(\frac{\pi x}{a}\right)\sin\left(\frac{\pi y}{b}\right).$$

**Figure 7.**
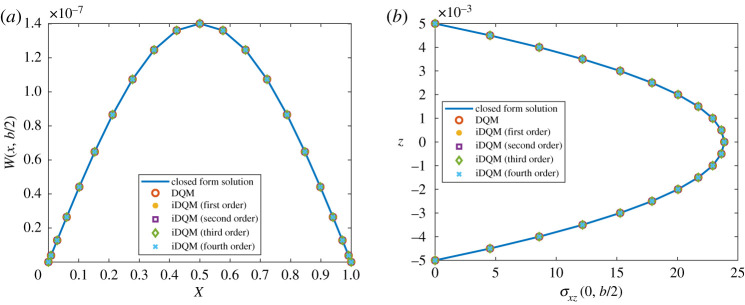
Plot of (*a*) deflection and (*b*) through-the-thickness shear stress, for isotropic plate. (Online version in colour.)

**Figure 8.**
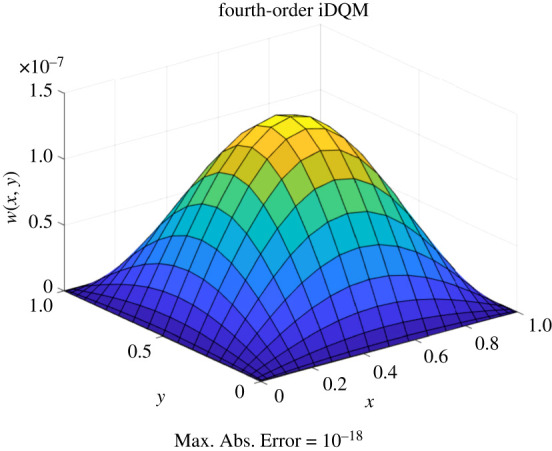
Transverse displacement *w* of the isotropic plate (21 × 21 grid). (Online version in colour.)

Solutions of the plate equation obtained by iDQM and DQM are shown in figure 7 where deflections and stresses for the plate match DQM and Navier’s solutions demonstrating accuracy of iDQM. Figure 8 shows the deformed planform of the plate under the loading by fourth-order iDQM along with the maximum absolute error in the entire domain. Furthermore, the percentage error of DQM and iDQM estimates in table 5 clearly demonstrates faster convergence for fourth-, third- and second-order iDQM over DQM, highlighting the computational merits of the proposed method.

**Table 5.** Percentage error of DQM and iDQM estimates for plate solution.

grid	first-order iDQM	second-order iDQM	third-order iDQM	fourth-order iDQM	DQM
*w*(*a*/2, *b*/2)					
5 × 5	$47$$.28$	$20$$.77$	$20$$.77$	$1$$.97$	$36$$.90$
7 × 7	$15$$.76$	$0$$.01$	$0$$.01$	$0$$.01$	$2$$.24$
11 × 11	$0$$.00$	$0$$.00$	$0$$.00$	$0$$.00$	$0$$.00$
*σ*_*xx*_(*a*/2, *b*/2, *h*/2)					
5 × 5	$22$$.97$	$9$$.52$	$9$$.52$	$1$$.35$	$25$$.64$
7 × 7	$7$$.33$	$0$$.01$	$0$$.01$	$0$$.01$	$1$$.41$
11 × 11	$0$$.00$	$0$$.00$	$0$$.00$	$0$$.00$	$0$$.00$
*σ*_*xz*_(0, *b*/2, 0)					
5 × 5	$14$$.30$	$6$$.55$	$6$$.55$	$0$$.04$	$15$$.75$
7 × 7	$3$$.99$	$0$$.24$	$0$$.24$	$0$$.01$	$1$$.21$
11 × 11	$0$$.14$	$0$$.00$	$0$$.00$	$0$$.00$	$0$$.00$

### (g) Error analysis (measure of numerical accuracy)

To appropriately examine the convergence of iDQM, it is important to assess the numerical accuracy of iDQM estimates subject to increased discretization of the domain. In this regard, we consider a BVP with the following set-up,

4.11 $$\begin{array}{rl} & \frac{{\text{d}}^{4}U}{\text{d}y^{4}}-2\frac{{\text{d}}^{2}U}{\text{d}y^{2}}+U=0,\;\forall \;y\;\in \;[0\;\;4]\\ \;\text{and}\; & U(y=0)=0,\;\frac{{\text{d}}^{2}U}{\text{d}y^{2}}{|}_{y=0}=2,\;U(y=1)=e,\;\frac{{\text{d}}^{2}U}{\text{d}y^{2}}{|}_{y=1}=3e. \end{array}\}$$

**Figure 9.**
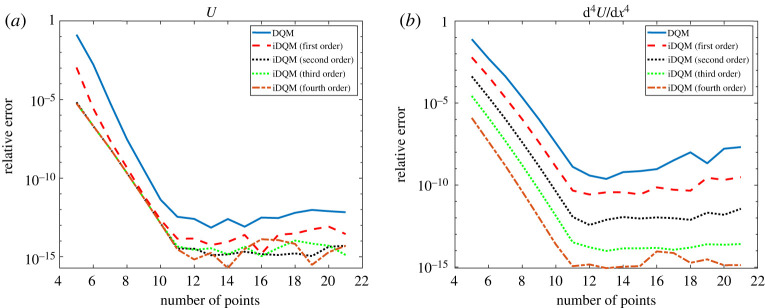
Relative error of BVP solutions for (*a*) *U* and (*b*) ∂^4^ *U*/∂*y*^4^. (Online version in colour.)

The exact solution of equation (4.11) is expressed as *U* = cosh *y* + cos *y*. After solving these equations using different iDQM schemes in accordance with the implementation procedures described in the appendix, the convergence of iDQM and DQM solutions for *U* and its higher derivatives based on Lagrange polynomial basis are shown in figure 9. According to figure 9, iDQM estimates provide improved convergence over DQM estimates in all cases considered. Clearly, DQM estimates show accumulation of error as the order of the numerical differentiation increases. This observation can be attributed to the perturbation of the total error caused by high-order derivatives of *M*_*N*_ (*y*), which increasingly affects the accuracy of DQM estimates especially at the boundary, subject to increase in *N*. On the other hand, iDQM estimates are less perturbed by a high-order integral of *M*_*N*_ (*y*), which varies inversely to a high order of *N* (as in *iDQM-by-integration*) or varies with *N* at a lesser rate than DQM (as in *iDQM-by-inversion*), leading to improved stability of the approximation error.

### (h) Error propagation (measure of numerical stability)

**Figure 10.**
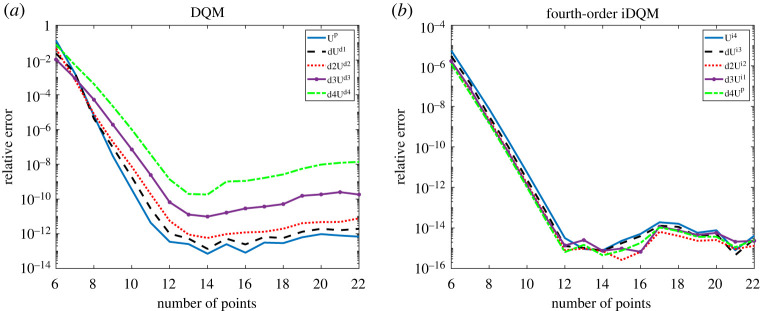
Error propagation of BVP solution based on Lagrange basis polynomial for (*a*) DQM estimate and (*b*) iDQM estimate. (Online version in colour.)

As a measure of numerical stability, propagated error arising from approximation of low-order functions from high-order estimates (by using iDQM) or approximation of high-order functions from lower-order estimates (by using DQM) is examined in this section. Figure 10 shows error propagation of the BVP example in §4.g, in which high-order functions (labelled superscript *d*) are computed from primary estimates (labelled superscript *p*) using DQM operation. On the other hand, low-order functions (labelled superscript *i*) are computed from primary estimates by *iDQM-by-inversion*.

According to figure 10, the errors resulting from high-order integration by *iDQM-by-inversion* are minimal, indicating numerical stability of iDQM operation. In the case of DQM, errors propagate by multiple orders for successive differentiation operation. While the error accumulation for low-order DQM approximations seems tolerable, the multiple order increment in the error due to high-order differentiation can cause inaccuracy which, in turn, leads to numerical instability. As already mentioned in §3.c, numerical stability of the DQM solution is significantly affected by the high-order derivative of *M*_*N*_ (*y*), which increases geometrically as the order of *N* increases. Therefore, on increasing *N*, high-order derivatives of *M*_*N*_ (*y*) quickly offset the total error to reach a lower error bound. However, the lower error bound of low-order estimates from high-order estimates remains stable after *iDQM-by-inversion*, indicating low error propagation and improved numerical stability.

## 5. Computational efficiency of the inverse differential quadrature method

To measure the computational efficiency of iDQM, we consider a comparison of the time and space memory requirements for the different benchmark problems to converge to a fixed value of the maximum absolute error *ϵ*_max_ for DQM and iDQM estimates. In this regard, the bandwidth (*b*) and the total primary degrees of freedom (*n*) of the final matrix **A** for a given algebraic system **Ax=b** are computed according to table 6.

**Table 6.** Computational efficiency of iDQM and DQM approximates.

time requirement (*nb*^2^)					
DQM	first-order iDQM	second-order iDQM	third-order iDQM	fourth-order iDQM	*ϵ*_max_
example (4g)					
3328	1859	1584	1584	1584	10^−13^
example (4c)					
320^a^	320	320	245	320	10^−13^
example (4d)					
12696	17 576	17 576	—	—	10^−15^
space requirement (*nb*)					
example (4g)					
208	143	132	132	132	10^−13^
example (4c)					
40^a^	40	40	35	40	10^−13^
example (4d)					
552	676	676	—	—	10^−15^

^a^ The DQM estimates converges to 10^−12^.

Some benchmark examples (4c, 4d and 4g) are chosen in table 6 to reflect different types of analysis, boundary conditions and nonlinearities that directly affect the computational complexities of a given numerical problem. According to table 6, iDQM approximation preserves the order of the numerical complexities of the problem in terms of time and space requirements as DQM. It is worth noting that the DQM estimate for example (4c) fails to converge to the threshold absolute maximum error *ϵ*_max_, i.e. 10^−13^. Thus, it is concluded that iDQM approximation preserves the computational efficiency of DQM approximation.

## 6. Conclusion

This study proposes a novel iDQM for numerical analysis of engineering systems. Given a system of high-order differential equations, the proposed iDQM approximates high-order variables rather than the original function which can be subsequently recovered by integration. To deal with issues bordering on computational inefficiency of analytical integration and numerical complexity of Gaussian integration, this study develops a novel strategy which relies on inversion of the existing formula of the DQM to compute the required iDQM weighting factors. Furthermore, to evaluate the performance of iDQM solutions, detailed derivations of iDQM error estimates based on integration and DQM inversion are developed in this work, which are then compared with DQM error estimates outlined in [23]. In the context of the iDQM scheme, two implementation approaches identified as Mixed iDQM and Full iDQM are proposed to obtain solutions of the examples provided in this work. Remarkably, the concept of Mixed iDQM provides an excellent opportunity to control the accuracy of system solutions by combining the numerical advantages of low-order differentiation and low-order integration to achieve an improved solution. Subsequently, a demonstration of iDQM implementation for functional approximation, and numerical solutions of systems of high-order ordinary differential equations and partial differential equations representing linear and nonlinear systems, prove that iDQM operations are potentially robust to furnish accurate solutions to numerical systems. Finally, an appraisal of the convergence and numerical stability of the iDQM approach suggests that, compared with DQM, improved convergence can be obtained for systems solution and improved numerical stability is guaranteed by using the proposed method without loss of computational efficiency.

## Data accessibility

This article has no additional data.

## Appendix A

**(a) One-dimensional inverse differential quadrature method discretization**

Consider a fourth-order ordinary differential equation,

A 1 $$\frac{{\text{d}}^{4}w}{\text{d}y^{4}}=q,$$

then the various numerical schemes in discretized form can be realized as follows:

**DQM**

A 2 $${\text{D}}^{\left(4\right)}\text{w}=\text{q},\;\text{w}={\left[w_{1},w_{2},\ldots ,w_{N}\right]}^{T},\;\text{q}={\left[q_{1},q_{2},\ldots ,q_{N}\right]}^{T},\;\text{w},\text{q}\in R^{N\times 1}$$

**First-order iDQM**

A 3 $$\begin{array}{rl} & {\text{D}}^{\left(3\right)}{\text{w}}^{\left(1\right)}=\text{q},\;\text{w}={\tilde{\text{D}}}^{\left(1\right)}{\tilde{\text{w}}}^{\left(1\right)},\;{\tilde{\text{w}}}^{\left(1\right)}={\left[{\text{w}}^{\left(1\right)}\;w(0)\right]}^{T}\\ \;\text{and}\; & {\tilde{\text{w}}}^{\left(1\right)}\in R^{(N+1)\times 1},\;{\tilde{\text{D}}}^{\left(1\right)}=[{\hat{\text{D}}}^{\left(1\right)}\;\text{I}],\;{\tilde{\text{D}}}^{\left(1\right)}\in R^{N\times (N+1)}. \end{array}\}$$

**Second-order iDQM**

A 4 $$\begin{array}{rl} & {\text{D}}^{\left(2\right)}{\text{w}}^{\left(2\right)}=\text{q},\;\text{w}={\tilde{\text{D}}}^{\left(2\right)}{\tilde{\text{w}}}^{\left(2\right)},\;{\tilde{\text{w}}}^{\left(2\right)}={\left[{\text{w}}^{\left(2\right)}\;w^{\left(1\right)}(0)\;w(0)\right]}^{T}\\ \;\text{and}\; & {\tilde{\text{w}}}^{\left(2\right)}\in R^{(N+2)\times 1},\;{\tilde{\text{D}}}^{\left(2\right)}=[{\hat{\text{D}}}^{\left(1\right)}\;\text{y}\;\text{I}],\;{\tilde{\text{D}}}^{\left(2\right)}\in R^{N\times (N+2)}. \end{array}\}$$

**Third-order iDQM**

A 5 $$\begin{array}{rl} & {\text{D}}^{\left(1\right)}{\text{w}}^{\left(3\right)}=\text{q},\;\text{w}={\tilde{\text{D}}}^{\left(3\right)}{\tilde{\text{w}}}^{\left(3\right)},\;{\tilde{\text{w}}}^{\left(3\right)}={\left[{\text{w}}^{\left(3\right)}\;w^{\left(2\right)}(0)\;w^{\left(1\right)}(0)\;w(0)\right]}^{T}\\ \;\text{and}\; & {\tilde{\text{w}}}^{\left(3\right)}\in R^{(N+3)\times 1},\;{\tilde{\text{D}}}^{\left(3\right)}=\left[{\hat{\text{D}}}^{\left(3\right)}\;\frac{{\text{y}}^{2}}{2}\;\text{y}\;\text{I}\right],\;{\tilde{\text{D}}}^{\left(3\right)}\in R^{N\times (N+3)}. \end{array}\}$$

**Fourth-order iDQM**

A 6 $$\begin{array}{rl} & {\text{I}}_{d}{\text{w}}^{\left(4\right)}=\text{q},\;\text{w}={\tilde{\text{D}}}^{\left(4\right)}{\tilde{\text{w}}}^{\left(4\right)},\;{\tilde{\text{w}}}^{\left(4\right)}={\left[{\text{w}}^{\left(4\right)}\;w^{\left(3\right)}(0)\;w^{\left(2\right)}(0)\;w^{\left(1\right)}(0)\;w(0)\right]}^{T},\\ \;\text{and}\; & {\tilde{\text{w}}}^{\left(4\right)}\in R^{(N+4)\times 1},\;{\tilde{\text{D}}}^{\left(4\right)}=\left[{\hat{\text{D}}}^{\left(3\right)}\;\frac{{\text{y}}^{3}}{6}\;\frac{{\text{y}}^{2}}{2}\;\text{y}\;\text{I}\right],\;{\tilde{\text{D}}}^{\left(4\right)}\in R^{N\times (N+4)}. \end{array}\}$$

All coefficient matrices used in this section are as defined in §2. ${\text{I}}_{d}\in R^{N\times N}$ is an identity matrix, *N* being the number of points chosen in the domain. **D**, $\tilde{\text{D}}$ matrix and **I** represent DQM and iDQM coefficients defined in §2.

**(b) Two-dimensional inverse differential quadrature method discretization**

Consider a second-order partial differential equation,

A 7 $$\frac{\partial^{2}w}{\partial x^{2}}+\frac{\partial^{2}w}{\partial y^{2}}=q,$$

then the various numerical schemes in discretized form can be realized as follows:

**DQM**

A 8 $$\begin{array}{rl} & {\text{D}}_{x}^{\left(2\right)}\text{w}{\text{I}}_{d}^{T}+{\text{I}}_{d}\text{w}{{\text{D}}_{y}^{\left(2\right)}}^{T}=\text{q}\\ \;\text{and}\; & \text{w}=\left[\begin{array}{ccc} w(x_{1},y_{1}) & \cdots & w(x_{1},y_{n})\\ \vdots & \ddots & \vdots \\ w(x_{n},y_{1}) & \ldots & w(x_{n},y_{n}) \end{array}\right],\;\text{w}\in R^{N\times N},\;{\text{D}}_{x}={\text{D}}_{y}=\text{D}. \end{array}\}$$

**First-order iDQM**

A 9 $$\begin{array}{rl} & {\tilde{\text{D}}}_{x}^{\left(1\right)}{\tilde{\text{w}}}^{\left(1\right)}{{\text{I}}_{\text{d}y}^{\left(1\right)}}^{T}+{\text{I}}_{\text{d}x}^{\left(1\right)}{\tilde{\text{w}}}^{\left(1\right)}{{\tilde{\text{D}}}_{y}^{\left(1\right)}}^{T}=\text{q},\;{\tilde{\text{w}}}^{\left(1\right)}\in R^{\left(N+1)\times (N+1\right)},\\ & {\tilde{\text{w}}}^{\left(1\right)}=\left[\begin{array}{cccc} w(0,0) & w_{y}(0,y_{1}) & \ldots & w_{y}(0,y_{n})\\ w_{x}(x_{1},0) & w_{xy}(x_{1},y_{1}) & \ldots & w_{xy}(x_{1},y_{n})\\ \vdots & \vdots & \ddots & \vdots \\ w_{x}(x_{n},0) & w_{xy}(x_{n},y_{1}) & \ldots & w_{xy}(x_{n},y_{n}) \end{array}\right],\\ \;\text{and}\; & {\tilde{\text{D}}}_{x}^{\left(1\right)}={\tilde{\text{D}}}_{y}^{\left(1\right)}=[\text{I}\;{\hat{\text{D}}}^{\left(1\right)}],\;{\text{I}}_{\text{d}x}^{\left(1\right)}={\text{I}}_{\text{d}y}^{\left(1\right)}=[\text{0}\;{\text{I}}_{d}] \end{array}\}$$

**Second-order iDQM**

A 10 $$\begin{array}{rl} & {\tilde{\text{D}}}_{x}^{\left(2\right)}{\tilde{\text{w}}}^{\left(2\right)}{{\text{I}}_{\text{d}y}^{\left(2\right)}}^{T}+{\text{I}}_{dx}^{\left(2\right)}{\tilde{\text{w}}}^{\left(2\right)}{{\tilde{\text{D}}}_{y}^{\left(2\right)}}^{T}=\text{q},\;{\tilde{\text{w}}}^{\left(2\right)}\in R^{\left(N+2)\times (N+2\right)},\\ & {\tilde{\text{w}}}^{\left(2\right)}=\left[\begin{array}{ccccc} w(0,0) & w_{y}(0,0) & w_{y^{2}}(0,y_{1}) & \ldots & w_{y^{2}}(0,y_{n})\\ w_{x}(0,0) & w_{xy}(0,0) & w_{xy^{2}}(0,y_{1}) & \ldots & w_{xy^{2}}(0,y_{n})\\ w_{x^{2}}(x_{1},0) & w_{x^{2}y}(x_{1},0) & w_{x^{2}y^{2}}(x_{1},y_{1}) & \ldots & w_{x^{2}y^{2}}(x_{1},y_{n})\\ \vdots & \vdots & \vdots & \ldots & \vdots \\ w_{x^{2}}(x_{n},0) & w_{x^{2}y}(x_{n},0) & w_{x^{2}y^{2}}(x_{n},y_{1}) & \ldots & w_{x^{2}y^{2}}(x_{n},y_{n}) \end{array}\right],\\ \;\text{and}\; & {\tilde{\text{D}}}_{x}^{\left(2\right)}={\tilde{\text{D}}}_{y}^{\left(2\right)}=[\text{I}\;\text{y}\;{\hat{\text{D}}}^{\left(2\right)}],\;{\text{I}}_{\text{d}x}^{\left(2\right)}={\text{I}}_{\text{d}y}^{\left(2\right)}=[\text{0}\;\text{0}\;{\text{I}}_{d}] \end{array}\}$$
